# Evaluation of an interleaved acquisition scheme for improved robustness of channel‐wise relative B_1_^+^ mapping at 7 T

**DOI:** 10.1002/mrm.70101

**Published:** 2025-09-29

**Authors:** Nico Egger, Laurent Ruck, Sophia Nagelstraßer, Judith Schirmer, Saskia Wildenberg, Andreas Bitz, Jürgen Herrler, Sebastian Schmitter, Michael Uder, Armin Michael Nagel

**Affiliations:** ^1^ Institute of Radiology University Hospital Erlangen, Friedrich‐Alexander‐Universität Erlangen‐Nürnberg (FAU) Erlangen Germany; ^2^ Electrical Engineering and Information Technology University of Applied Sciences ‐ FH Aachen Aachen Germany; ^3^ Siemens Healthcare Erlangen Germany; ^4^ Physikalisch‐Technische Bundesanstalt (PTB) Braunschweig and Berlin Germany; ^5^ Center for Magnetic Resonance Research University of Minnesota Minneapolis Minnesota USA; ^6^ Division of Medical Physics in Radiology German Cancer Research Centre (DKFZ) Heidelberg Germany

**Keywords:** 7 Tesla, B1+ mapping, heart, parallel transmission, prostate, ultra‐high field

## Abstract

**Purpose:**

The purpose was to evaluate, whether an interleaved acquisition scheme for a fast relative $B_{1}^{+}$ mapping method at 7 T reduces the likelihood of errors from exceeding the linear flip angle (FA) regime compared to a conventional sequential acquisition.

**Methods:**

Simulations of a channel‐wise relative $B_{1}^{+}$ mapping sequence were performed for sequential and interleaved acquisition schemes at different reference voltages (≙ different FAs). The simulations were performed for a phantom, the heart and the prostate and were based on 7 T ground‐truth (GT) $B_{1}^{+}$ data acquired with an actual FA imaging sequence (phantom) or obtained from electromagnetic field simulations (heart/prostate). Acquisition schemes were evaluated based on their signal linearity by calculating a normalized mean FA error between simulated signal intensities and GT $B_{1}^{+}$ data. Additionally, validation measurements of relative $B_{1}^{+}$ maps with the sequential and interleaved acquisition schemes were acquired for the phantom.

**Results:**

Validation measurements showed a good agreement with the simulation results for both acquisition schemes and displayed stronger deviations to the GT $B_{1}^{+}$ data for the sequential scheme. The quantitative evaluation yielded higher FA errors for the sequential acquisition scheme for all three regions and all simulated reference voltages. At the same level of error, mean signals were higher for the interleaved acquisition scheme in all cases. Differences between interleaved and sequential acquisition schemes were most pronounced in the steady‐state.

**Conclusion:**

An interleaved acquisition of channel‐wise relative $B_{1}^{+}$ maps extends the range of the linear FA regime, reducing the likelihood of errors and increasing the robustness of the approach.

## 1 INTRODUCTION

To counteract excitation non‐uniformities^1^ caused by the shortened RF wavelength of the excitation field ($B_{1}^{+}$) at ultra‐high magnetic field strengths (UHF), parallel transmission (pTx) has been introduced, using multiple independent transmit elements.^2^, ^3^, ^4^ Through a superposition of the individual fields, the composite $B_{1}^{+}$ field can be optimized to mitigate flip angle (FA) inhomogeneities. However, this optimization requires knowledge of the spatial distribution of the individual transmit fields. Because the field distributions are subject‐specific and also depend on coil positioning, designing tailored pTx pulses typically relies on measuring channel‐wise $B_{1}^{+}$ maps. Although many different methods exist for this purpose,^5^ acquiring $B_{1}^{+}$ maps for each individual transmit channel can lead to time intensive measurements.

In this context, Van de Moortele et al.^6^ introduced a method that, despite only yielding relative $B_{1}^{+}$ values (biased by the square root of the proton density), has become popular because of its rapid channel‐wise acquisition. The method relies on gradient echo (GRE) measurements in the small FA regime with negligible T_1_‐weighting to ensure a linear relationship between the measured signal intensities and corresponding $B_{1}^{+}$ magnitudes. In 2D applications, the TR can typically be set long enough to avoid T_1_‐weighting. However, in 3D acquisitions with large FOVs (e.g., UHF body imaging), feasible acquisition times necessitate much shorter TRs, often below 10 ms.^7^, ^8^ In such cases, non‐negligible T_1_‐weighting may restrict the range of the linear FA regime. For instance, with a TR of 5 ms and myocardial T_1_ time of approximately 2 s at 7 T,^9^ calculations based on the FLASH equation show that deviations from linearity already reach approximately 24% for FAs of only 2°. Combined with the large dynamic range of the excitation field,^10^, ^11^, ^12^ this makes UHF body imaging particularly susceptible to exceeding the linear FA regime, introducing errors and reducing the accuracy of the $B_{1}^{+}$ maps.

Contributing to this issue is the typically sequential acquisition scheme^6^, ^13^ of the relative $B_{1}^{+}$ mapping sequence, where GRE measurements are acquired sequentially for each element of the transmit coil. With this approach, the FA distribution is identical between each sequence repetition and T_1_ relaxation is limited by the TR. A better alternative might be an acquisition scheme where the k‐space data for each transmit channel are acquired in an interleaved fashion,^13^ altering the excitation with each TR.

The aim of this work was to investigate quantitatively, whether such an adapted acquisition scheme improves the signal linearity of relative $B_{1}^{+}$ maps compared to the conventional approach. For this purpose, simulations were performed for phantom, heart and prostate data and validation measurements were acquired in the phantom and in vivo. Simulations were evaluated quantitatively by calculating the normalized mean FA error between the simulated signals and ground‐truth (GT) FA data.

## 2 METHODS

### 2.1 Channel‐wise relative $B_{1}^{+}$ mapping method

The channel‐wise relative $B_{1}^{+}$ mapping approach is based on a series of GRE acquisitions in the small FA regime.^6^ The series consist of $N_{\mathrm{Tx}}$ measurements, where $N_{\mathrm{Tx}}$ is the number of available transmit (Tx) channels of which only one channel is used for excitation during each of the acquisitions. The images are acquired with all $N_{\mathrm{Rx}}$ receive (Rx) channels and provided that FAs are small and T_1_‐weighting is negligible (i.e., in the linear FA regime), the signal intensities $S_{m,n}\left(r\right)$ of Rx channel m and Tx channel n are given by:

(1) $$\left|S_{m,n}\left(r\right)\right|=c\;\left|B_{1,m}^{-}\left(r\right)\right|\;\left|B_{1,n}^{+}\left(r\right)\right|\;\rho_{0}\left(r\right),$$

with a complex scaling factor c, the channel‐wise receive and transmit fields $B_{1,m}^{-}\left(r\right)$ and $B_{1,n}^{+}\left(r\right)$ and the proton density $\rho_{0}\left(r\right)$. Based on the measured signal intensities, estimations of the channel‐wise relative $B_{1}^{+}$ magnitudes $\left|{\hat{B}}_{1,n}^{+}\left(r\right)\right|$ can be calculated via^6^:

(2) $$\left|{\hat{B}}_{1,n}^{+}\left(r\right)\right|=\frac{\sum_{m}^{N_{\mathrm{Rx}}}\left|S_{m,n}\left(r\right)\right|}{\sqrt{\sum_{m}^{N_{\mathrm{Rx}}}\sum_{n}^{N_{\mathrm{Tx}}}\left|S_{m,n}\left(r\right)\right|}}.$$

Note that these $B_{1}^{+}$ estimations are biased by the square root of $\rho_{0}\left(r\right)$ as the proton density is set to 1 during the derivation of Eq. (2).^6^ Furthermore, we assume that the sum of magnitudes over the Rx channels is equal to that over the Tx channels.^6^ For the RF coil used in this study, this assumption was validated in a previous publication^8^ (Supporting Information), demonstrating good agreement.

It is important to note that the above equations are only valid under the assumption of a linear relationship between the measured signal and the $B_{1}^{+}$ magnitude. Deviations from the linear FA regime will, therefore, introduce additional errors in the $B_{1}^{+}$ estimations.

### 2.2 Acquisition schemes

**FIGURE 1:**
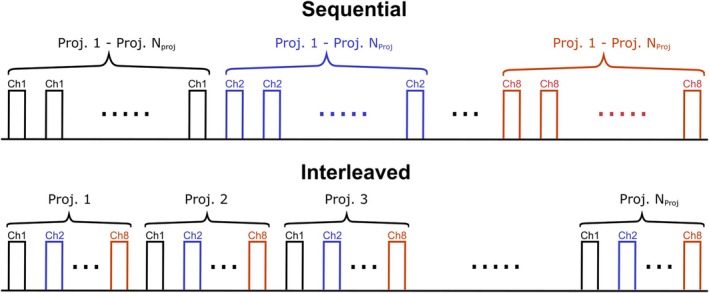
Illustration of the sequential (top) and interleaved (bottom) acquisition schemes used for the channel‐wise relative $B_{1}^{+}$ mapping approach. For the conventional sequential acquisition, all of the $N_{\text{Proj}}$ k‐space projections (or k‐space lines) are acquired for a fixed transmit channel, before repeating the measurement with the next transmit channel. In contrast, for the interleaved acquisition scheme, the same k‐space projection is acquired consecutively for each transmit channel before moving on to the next k‐space projection. This process is repeated $N_{\text{Proj}}$ times and in total the same k‐space information is acquired as with the sequential acquisition scheme.

For the series of GRE images, k‐space data is conventionally acquired using a sequential acquisition scheme,^6^, ^13^ where all k‐space projections/lines are measured for one Tx channel before repeating the process for the subsequent channels. As an alternative, we investigated an interleaved acquisition scheme,^13^ in which the same k‐space projection is acquired consecutively for each Tx channel before moving to the next k‐space projection. Both acquisition schemes are illustrated in Figure 1.

### 2.3 Data simulation

Simulations of the relative $B_{1}^{+}$ acquisition schemes were performed for phantom, heart, and prostate data. The phantom consisted of four stacked disks with two outer disks filled with a NaCl solution and two inner disks filled with oil (see Figure S1). Note that the phantom simulations and evaluations in this work were limited exclusively to the outer disks filled with the NaCl solution.

For the phantom, the simulations were based on single‐channel transmit efficiencies $B_{1,n}^{+,\eta }\left(r\right)$ (in μT/V) obtained from measurements with an actual flip angle imaging^14^ (AFI) sequence (TR_1_/TR_2_ = 15/75 ms, TE = 3.03 ms, $\alpha_{\mathrm{nom}}$ = 70°, resolution = (4 mm)^3^, randomized x‐y‐spoiling,^15^ projections = 6000 × 8, acquisition time = 8 × 9 min) using an 8Tx/16Rx body array (Rapid Biomedical, Rimpar, Germany) (see Figure S1). For the heart and prostate, simulations of the relative $B_{1}^{+}$ acquisition schemes were also based on single‐channel transmit efficiencies of the 8Tx/16Rx body array. However, in this case, the transmit efficiencies were obtained from electromagnetic field simulations of the Duke body model.^16^

The transmit efficiencies were used to calculate channel‐wise GT FA distributions $\alpha_{n}\left(r\right)$ at different reference voltages $U_{\mathrm{ref}}$, based on:

(3) $$\alpha_{n}\left(r,U_{\mathrm{ref}}\right)=\gamma {\cdot B}_{1,n}^{+,\eta }\left(r\right)\cdot \frac{\alpha_{\mathrm{nom}}}{180^{{}^{\circ}}}\cdot 1\;\mathrm{ms}\cdot U_{\mathrm{ref}}.$$

Assuming a square pulse and using a nominal FA $\alpha_{\mathrm{nom}}$ of 8°.

Taking the FA distributions as GT, the magnitudes of the longitudinal magnetization $M_{z}\left(r\right)$ and transverse magnetization $M_{\mathrm{xy}}\left(r\right)$ were simulated iteratively for the pulse sequence of the relative $B_{1}^{+}$ mapping method at different reference voltages. Each iteration corresponds to one sequence repetition and consists of two steps:

(4) $$\begin{array}{cc} \left(1\right)\;M_{z}^{\left(\mathrm{new}\right)}\left(r\right) & =1+\left(M_{z}^{\left(\mathrm{old}\right)}\left(r\right)\cdot \cos\left(\alpha_{n}\left(r,U_{\mathrm{ref}}\right)\right)-1\right)\cdot e^{-\frac{\mathrm{TR}}{T_{1}}}\\ \left(2\right)\;M_{\mathrm{xy}}^{\left(\mathrm{new}\right)}\left(r\right) & =M_{z}^{\left(\mathrm{old}\right)}\left(r\right)\cdot \sin\left(\alpha_{n}\left(r,U_{\mathrm{ref}}\right)\right), \end{array}$$

where old denotes the magnetization of the previous and new the magnetization of the current iteration ($M_{z}^{\left(0\right)}\left(r\right)=1$). The TR was set to 4.5 ms, corresponding to realistic values used for 3D relative $B_{1}^{+}$ acquisitions.^7^, ^8^ For the longitudinal relaxation times, literature values of 1925 ms^9^ and 1800 ms^17^ were used for the heart and prostate, respectively. For the phantom, the T_1_ value of the NaCl solution was determined experimentally to be 2044 ms.

The simulations of the examined acquisition schemes differed in their iteration approach (see Figure 1). For the sequential scheme, simulations were performed separately for each Tx channel, with a fixed channel number n for all iterations. For the interleaved scheme, the Tx channel number n cycled between 1 and 8, changing with each iteration. For both acquisition schemes, the steps were repeated until a steady‐state was reached and the transversal magnetization of the last iteration step was treated as the measured signal intensity.

In addition to the steady‐state simulations, transient‐state simulations were performed. In contrast to the steady‐state approach, these simulations were not run until converging to a constant state. Instead, the longitudinal and transverse magnetizations were computed for a fixed number of iteration steps (≙ sequence repetitions), and the resulting transverse magnetizations were averaged across all iterations to estimate the measured signal intensity instead of using only the final step. For the sequential acquisition, the simulation was adapted such that the final magnetization state from the previous channel served as the initial state for the subsequent channel. The transient‐state simulations were performed for both acquisition schemes and for different numbers of fixed iteration steps.

### 2.4 Evaluation of simulation data

For the evaluation of the simulation results, a linear fit between the simulated signal intensities and the corresponding GT FAs was determined (minimizing the mean absolute error). The signal intensities were scaled to FA values using the slope of the linear fit and absolute deviations from the GT FAs were calculated for each voxel to quantify the errors. The FA errors were then averaged over all voxels within the region of interest (ROI) and normalized by the mean of the GT FAs (to enable a comparison between different reference voltages), yielding normalized mean FA errors $\bar{\Delta \mathrm{FA}}$. For the phantom, the ROI included all voxels in the NaCl disks with non‐zero FAs of the single‐channel AFI acquisitions. For the heart and prostate, the corresponding organ ROI of the body model was used. In addition to the FA errors, mean signals within the ROI were also calculated for every simulation.

### 2.5 Validation measurements

To validate the simulation results, channel‐wise relative $B_{1}^{+}$ maps of the phantom were acquired with the sequential and interleaved acquisition schemes. Both schemes were implemented for a density‐adapted 3D‐radial^18^ full‐projection implementation of the relative $B_{1}^{+}$ mapping method. Measurements were carried out on a 7 T whole‐body MR‐system (MAGNETOM Terra.X, Siemens Healthcare, Erlangen, Germany) using the 8Tx/16Rx body array. The $B_{1}^{+}$ maps were acquired for the phantom with following sequence parameters: TR = 4.5 ms, TE = 2.02 ms, $\alpha_{\mathrm{nom}}$ = 8°, $T_{P}$ = 500 μs, resolution = (4 mm)^3^, projections = 6000, acquisition time = 3:36 min, and reference voltages of 25, 50, 75, 100, and 150 V. For each reference voltage, measurements were acquired with both acquisition schemes.

## 3 RESULTS

### 3.1 Validation measurements

**FIGURE 2:**
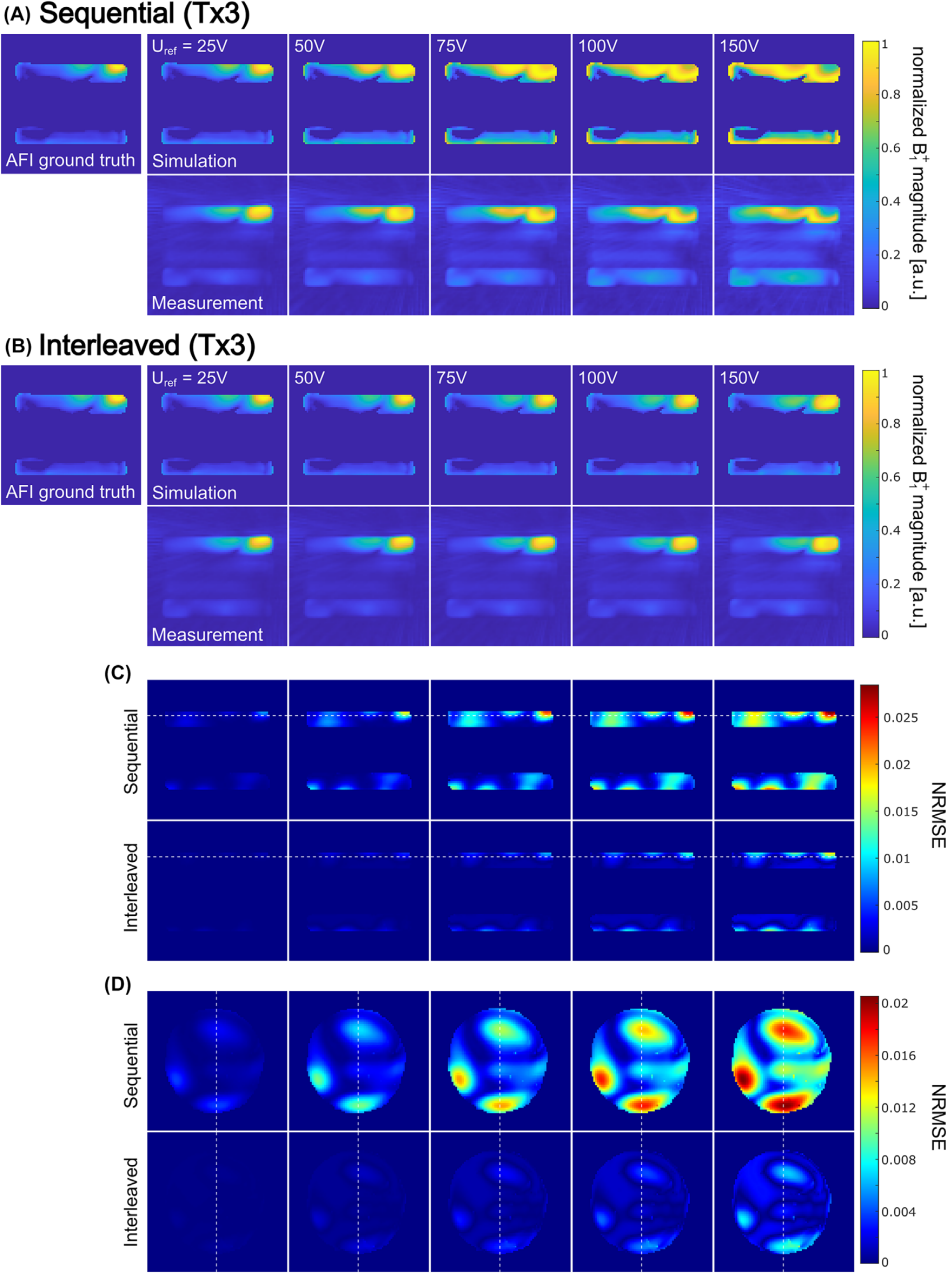
(A,B) Actual flip angle imaging (AFI) ground‐truth (GT) $B_{1}^{+}$ maps and corresponding simulated (top) and measured (bottom) relative $B_{1}^{+}$ magnitudes for the disk phantom (see Figure S1). Note that the oil‐filled disks in the middle were not used in the evaluation and were masked in the AFI GT and simulations. Data are shown for different reference voltages (≙ flip angles) for sequential (A) and interleaved (B) acquisitions and were normalized to the maximum within the slice. At low voltages, sequential acquisitions agree well with the GT, but deviations increase at higher voltages. In contrast, interleaved acquisitions maintain good agreement across all voltages. In general, a good match between simulations and measurements can be observed. (C,D) Normalized root mean square error (NRMSE) maps between GT and simulations for a transversal (C) and coronal (D) slice. Errors were averaged across transmit channels and normalized by the reference voltage. Both schemes show spatially heterogeneous NRMSE that increases with voltage, but the interleaved scheme consistently yields lower errors than the sequential scheme.

Figure 2 depicts $B_{1}^{+}$ magnitudes (Tx3), acquired/simulated for the phantom with the sequential (A) and interleaved (B) acquisition schemes at different reference voltages. In general, a good match between the simulations and the validation measurements can be observed. For the lowest reference voltage, the simulated $B_{1}^{+}$ magnitudes for both acquisition schemes show good agreement to the GT. For increasing voltages, strong deviations to the GT become visible for the sequential acquisition scheme. In contrast, the distribution of the $B_{1}^{+}$ magnitude appears more stable for the interleaved acquisition scheme, with comparably small deviations to the GT. Accordingly, the normalized root mean square error maps included in Figure 2C,D exhibit consistently higher errors for the sequential scheme than for the interleaved scheme (single channel error maps are shown in Figure S2).

For a validation in vivo, Figure S3 shows a comparison between relative $B_{1}^{+}$ magnitudes for both acquisition schemes to an AFI GT measurement. The interleaved scheme generally exhibits a higher correlation to the AFI GT, and correlation degrades more rapidly with increasing reference voltage for the sequential scheme.

### 3.2 Evaluation of the signal linearity

**FIGURE 3:**
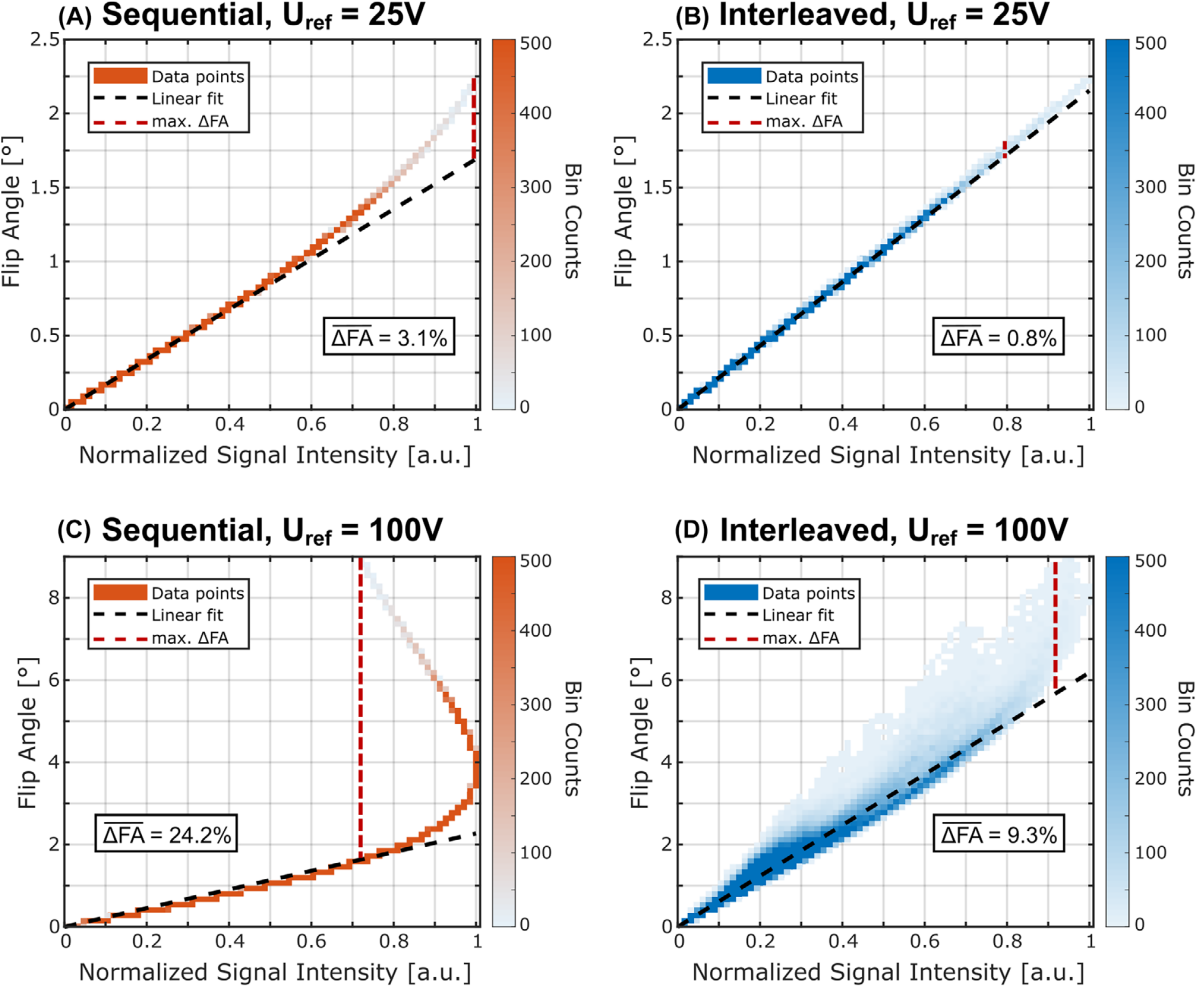
Binned scatter plots of the ground‐truth flip angle (FA) over the normalized signal intensity resulting from simulations of the phantom with the sequential (A,C) and interleaved (B,D) acquisition scheme. The data comprises the 3D region of interest (ROI) covering the upper and lower NaCl disks and is shown for a low (25 V) and a high (100 V) reference voltage (additional voltages are shown in Figure S4). The plot depicts a histogram of the data point distribution, with the bin count indicated by the colorbar. Because the simulations were performed for the steady‐state, the data of the sequential acquisition scheme follow the analytical solution of the FLASH equation. For a reference voltage of 25 V, the data points of both acquisition schemes are distributed close to the linear fit (black line), with slightly more deviations for the sequential scheme. Maximum FA errors (red line) are 0.54° for the sequential and 0.09° for the interleaved scheme. For a reference voltage of 100 V, deviations increase in both cases, with maximum FA errors of 7.3° for the sequential and 3.2° for the interleaved scheme. Normalized mean FA errors $\bar{\Delta \mathrm{FA}}$ are specified in each plot and are considerably smaller for the interleaved acquisition scheme.

In Figure 3, evaluations of the phantom simulations with two reference voltages (25 V, 100 V, additional voltages shown in Figure S4) are shown for the sequential (Figure 3A,C) and interleaved (Figure 3B,D) acquisition schemes. The data from the 3D ROI covering the upper and lower NaCl disks are depicted as a binned scatter plot between the GT FA and the simulated normalized signal intensity. For a reference voltage of 25 V, the data points of both acquisition schemes are distributed close to the linear fit. Data points deviating from the linear fit are visible for the sequential acquisition scheme at high FAs, although with a relatively low bin count. The maximum FA error is 0.54° for the sequential scheme and 0.09° for the interleaved scheme. For a reference voltage of 100 V, data bins with strong deviations to the linear fit are visible for both acquisition schemes. Nevertheless, for the interleaved scheme, the bin counts in areas with strong deviations are comparably low, whereas a high density close to the linear fit is visible. With the higher reference voltage, the maximum FA error increases to 7.3° for the sequential scheme and 3.2° for the interleaved scheme. For both voltages, the normalized mean FA error $\bar{\Delta \mathrm{FA}}$ is lower for the interleaved scheme than for the sequential scheme (0.8% vs. 3.1% and 9.3% vs. 24.2%).

**FIGURE 4:**
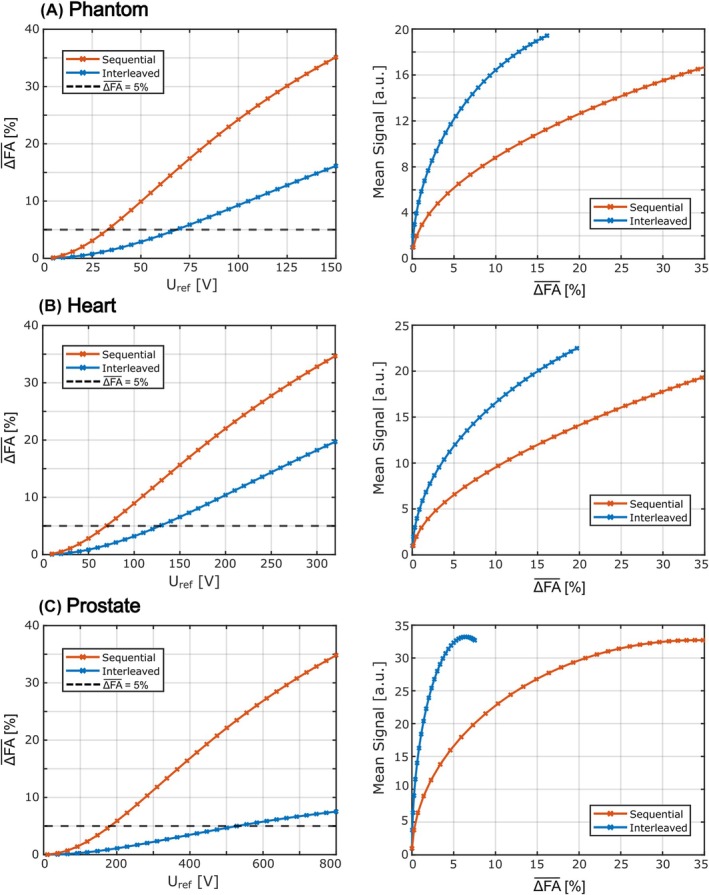
$\bar{\Delta \mathrm{FA}}$ over the reference voltage (left) and mean signals over $\bar{\Delta \mathrm{FA}}$ (right) for simulations of the sequential and interleaved acquisition schemes for data of the phantom (A), heart (B), and prostate (C). Corresponding plots of the mean signal over the reference voltage are depicted in Figure S5. For the three data sets, a similar trend for $\bar{\Delta \mathrm{FA}}$ can be observed, with increasing errors for increasing reference voltages. However, in all simulated cases, $\bar{\Delta \mathrm{FA}}$ values are lower for the interleaved acquisition than for the sequential acquisition. For the mean signals over $\bar{\Delta \mathrm{FA}}$, the trend is also similar for all three data sets, with higher values for the interleaved acquisition scheme in all cases.

Figure 4 (left column) shows $\bar{\Delta \mathrm{FA}}$ values for different reference voltages for the phantom (Figure 4A), heart (Figure 4B) and prostate simulations (Figure 4C). In general, the errors increase with higher reference voltages for both acquisition schemes. In all simulated cases, $\bar{\Delta \mathrm{FA}}$ is smaller for the interleaved scheme, allowing for higher reference voltages at the same error. The reference voltages that lead to the same $\bar{\Delta \mathrm{FA}}$ of approximately 5% are 35 V (sequential) and 70 V (interleaved) for the phantom, 70 V (sequential) and 130 V (interleaved) for the heart and 174 V (sequential) and 528 V (interleaved) for the prostate simulations. Consequently, the mean signals over $\bar{\Delta \mathrm{FA}}$ (right column) yield higher values for the interleaved acquisition scheme. Corresponding plots of the mean signal over the reference voltage are depicted in Figure S5. Additionally, $B_{1}^{+}$ magnitude maps of the heart and prostate are shown in Figure S6 for simulations of both acquisition schemes.

### 3.3 Transient‐state simulations

**FIGURE 5:**
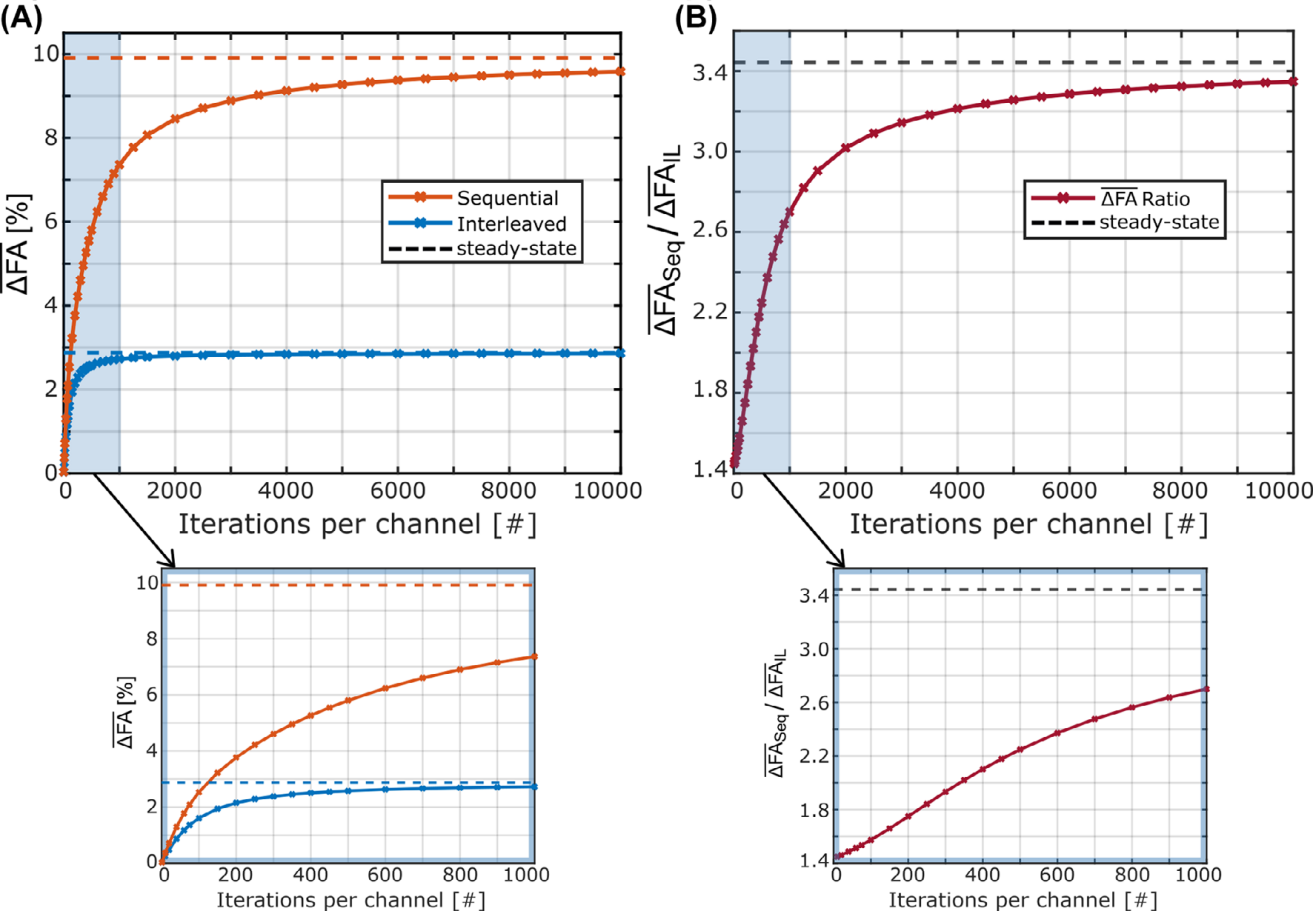
$\bar{\Delta \mathrm{FA}}$ (A) and its ratio between sequential and interleaved acquisition (B) in relation to the number of iterations (≙ sequence repetitions) per channel for transient‐state simulations of the phantom (U_ref_ = 50 V). For these simulations, the magnetizations of each iteration step were summed up and averaged over the total number of iterations instead of only using the magnetization of the last iteration step. The $\bar{\Delta \mathrm{FA}}$ values and ratios obtained in the steady‐state are represented by the dashed lines. For both acquisition schemes, the errors increase with more repetitions and asymptotically approach the steady‐state values. For the interleaved acquisition, the steady‐state values are reached more quickly than for the sequential acquisition. Accordingly, the ratio between the errors of both acquisition schemes increases with a higher number of iterations, growing from approximately 1.4 at the lowest number of simulated iterations (10) to approximately 3.4 in the steady state. Notably, $\bar{\Delta \mathrm{FA}}$ values are smaller for the interleaved acquisition in all cases.

Results of transient‐state simulations are depicted in Figure 5 for simulations of the phantom with $U_{\mathrm{ref}}$ = 50 V. The simulations were performed for different numbers of iterations per channel, and $\bar{\Delta \mathrm{FA}}$ was calculated. For both acquisition schemes, the errors increase with more iterations, converging toward the steady‐state values. For the interleaved acquisition scheme, the corresponding steady‐state value is reached faster than for the sequential acquisition. For 6000 iterations, corresponding to the number of projections used for the validation measurements, $\bar{\Delta \mathrm{FA}}$ values were 9.4% for the sequential scheme (steady‐state = 9.9%) and 2.9% for the interleaved scheme (steady‐state = 2.9%). For a lower number of iterations, the differences between both acquisition schemes are considerably smaller than in the steady‐state. Nevertheless, errors are higher for the sequential scheme for all simulated cases.

## 4 DISCUSSION

In this work, we evaluated, whether an interleaved acquisition scheme for a commonly used channel‐wise relative $B_{1}^{+}$ mapping method^6^ at UHF mitigates the risk of exceeding the linear FA regime. As originally published by Van de Moortele,^6^ the relative $B_{1}^{+}$ mapping method relies on acquisitions that are both in the small FA regime and also exhibit minimal T_1_‐weighting to ensure a linear relationship between measured signals and corresponding FAs. For 3D applications, however, the short TRs needed to facilitate feasible acquisition times can lead to a T_1_‐weighting that is no longer negligible. In combination with the large dynamic range of the RF field, deviations from the linear relationship because of high FAs have been identified as a potential source of errors in previous publications at 7 T.^8^

Methods to combat this issue have recently been published,^10^, ^11^ relying on the acquisition of additional $B_{1}^{+}$ maps at different voltages. An interleaved acquisition for relative $B_{1}^{+}$ mapping has initially been proposed by Brunheim et al.^13^ for 7 T UHF body imaging and was also used previously by de Buck et al.^11^ and Kent et al.^19^ Although the former study used the interleaved acquisition to reduce measurement time, the latter studies used the interleaved acquisition scheme to mitigate magnetization history effects from T_1_. However, neither study included a quantitative comparison to the conventional sequential acquisition, and the use of an interleaved acquisition scheme in combination with the Van de Moortele approximation has not yet been evaluated.

For the quantitative comparison of the sequential and interleaved acquisition schemes performed in this work, evaluations were primarily based on simulations. These simulations consisted of excitation and relaxation effects and did not include more complex processes such as spoiling, which represents a limitation of this work. Nevertheless, validation measurements—including one in vivo dataset (Figure S3)—showed a good agreement with the simulation data. The quantitative evaluation was based on a linear fit between the simulated signals and the GT FA and a calculation of the normalized mean FA error. The evaluation was performed this way since it is comparable to the commonly used approach of scaling the relative $B_{1}^{+}$ maps to absolute values.^7^

In terms of the signal linearity, the interleaved acquisition scheme was found to be superior to the sequential scheme, with lower errors regardless of the reference voltage used for the simulation. A possible explanation for this is that voxels close to one Tx channel experience a comparatively high FA excitation when measuring with this channel, but a smaller FA excitation during the subsequent measurements with the other channels, because of the limited overlap of their transmit fields. Compared to a repeated excitation with one fixed Tx channel, the interleaved acquisition, therefore, better facilitates the relaxation of the magnetization toward equilibrium, which in turn increases the range of the linear regime.

The main limitation of this work is the fact that the evaluation was only shown for a single RF coil setup, with other setups potentially yielding different results. However, the main difference of other RF coil setups would be different overlaps between the transmit profiles, which is partially covered by the different locations of the phantom, heart, and prostate, evaluated in this study. Additionally, preliminary evaluations based on GT AFI measurements of a 4Tx/16Rx 7 T breast coil showed similar results, with a superior performance of the interleaved acquisition scheme (see Figure S7). Different Tx channel orderings for the interleaved acquisition scheme were also analyzed for the 8Tx/16Rx body array (see Figure S8), but only showed negligible differences.

Another limitation is that the T_1_ times examined in this study were all in the upper range of physiologically expected values. However, an extended analysis across the expected in vivo T_1_ range showed that the interleaved scheme consistently yields lower errors across different combinations of T_1_ values and reference voltages (see Figure S9).

As a topic for future investigation, the influence of the acquisition scheme on the point spread function is of interest, because it may affect the effective resolution between interleaved and sequential approaches. This aspect was not assessed here, because our simulations were not k‐space‐based and did not assume a specific trajectory. Furthermore, in pTx applications, absolute $B_{1}^{+}$ quantification per Tx channel is often required. This is typically achieved by scaling relative $B_{1}^{+}$ maps with one or more additional absolute calibration scans.^7^, ^13^, ^20^ Current research aims to accelerate the acquisition of such absolute reference data^21^ and improve accuracy for applications with large dynamic $B_{1}^{+}$ ranges.^12^, ^22^

Although much of the quantitative evaluation in this study was based on steady‐state assumptions, transient‐state simulations showed that differences in error between sequential and interleaved acquisitions decrease with fewer excitations. Therefore, the benefits of an interleaved scheme are expected to be less pronounced for slice‐selective than for non‐selective acquisitions. Nevertheless, even for slice‐selective measurements (excitations <100), our evaluation yielded errors more than 40% higher with the sequential scheme. Depending on sequence parameters (e.g., minimal TR), single‐slice acquisitions could still benefit substantially from an interleaved approach. However, as slice‐interleaving increases the effective TR, the influence of the acquisition scheme is expected to diminish as the number of slices increases.

Nevertheless, although many current calibration strategies rely on multi‐slice acquisitions, 3D approaches may offer advantages such as higher SNR efficiency and reduced slice profile effects. Their suitability for higher acceleration factors^19^, ^23^ and improved motion robustness, especially when combined with non‐Cartesian sampling,^7^, ^8^ makes them particularly effective for body imaging at UHF. For 3D relative $B_{1}^{+}$ acquisitions, especially with large FOVs,^7^, ^8^, ^24^, ^25^, ^26^, ^27^ reduced T_1_‐weighting and the resulting extended linear FA range favor an interleaved scheme over a sequential acquisition scheme.

## 5 CONCLUSION

In this work, we evaluated whether an interleaved acquisition scheme for a relative $B_{1}^{+}$ mapping method provides a more robust approach in terms of signal linearity compared to a conventional, sequential acquisition. The results indicate that the interleaved acquisition scheme extends the range of the linear FA regime and, therefore, mitigates the risk of potential errors, enhancing the accuracy of the approach. Based on the reported results and because of its straightforward implementation, we generally recommend the use of the interleaved scheme over the sequential scheme for the acquisition of relative $B_{1}^{+}$ maps.

## CONFLICT OF INTEREST STATEMENT

J.H. is an employee of Siemens Healthcare. A.N. receives research support from Siemens Healthcare.

## Supporting information


**Figure S1.** (A) Structure of the 8Tx/16Rx body array used in this work. The RF array consists of an upper and a lower part with 4Tx/8Rx channels each. In the transmit case, two of the receive elements (numbered 1–16) are combined to one transmit element (colored). (B) Illustration and measurement setup of the phantom used in this work. The phantom consists of two disks filled with 2 L of a NaCl solution and two disks filled with 2 L of oil (height ∼35 mm each, diameter ∼240 mm). For the measurement, the disks are stacked on top of each other, with the two NaCl disks on the outside and the oil disks positioned between them. Note that for the simulations and evaluations performed in this work, only the data from the NaCl disks were analyzed.
**Figure S2.** Channel‐wise relative absolute errors between the phantom simulations of the sequential (left) and interleaved (right) acquisition schemes and the AFI ground‐truth (GT) for a reference voltage of 150 V. The errors are given relative to the FA of the GT and are depicted for a transversal and coronal slice (indicated by white dashed line). For the sequential acquisition scheme, errors are highest in areas close to the transmitting element, whereas for the interleaved scheme the errors distribution appears similar between different transmit channels. In general, errors are considerably higher for the sequential acquisition scheme.
**Figure S3.** (A) AFI ground‐truth (GT) and corresponding relative $B_{1}^{+}$ maps for Tx1, acquired with the sequential and interleaved acquisition schemes for three different reference voltages in an in vivo measurement of the heart. The $B_{1}^{+}$ maps were normalized to the maximum within the slice to ensure consistent windowing and a 3D mask was generated based on the AFI measurement. (B) Correlation factors between the AFI GT and the acquired relative $B_{1}^{+}$ maps based on all data points within the 3D mask volume. For the lowest reference voltage of 50 V the relative $B_{1}^{+}$ maps of both acquisition scheme show a good qualitative (A) and quantitative (B) agreement with the GT. With increasing voltage, the correlation only decreases moderately for the interleaved scheme, whereas it drops sharply for the sequential scheme. Correspondingly, the qualitative evaluation shows large deviations to the GT at 300 V for the sequential scheme, whereas a better match is observed for the interleaved scheme. Relative $B_{1}^{+}$ sequence parameters as described in section 2.5, with 10 000 projections. AFI parameters were: TR1/TR2/TE = 15/75/3.03 ms, nominal FA = 65°, resolution = (4 mm)^3^, projections = 5000, acquisition time = 7:30 min.
**Figure S4.** Extension of the scatter plots shown in Figure 3 for four additional reference voltages. The plots depict histograms of the data point distribution of the ground‐truth FA over the normalized signal intensity resulting from simulations of the phantom with the sequential (left) and interleaved (right) acquisition schemes. In addition, the maximum FA deviations are depicted and the normalized mean FA errors $\bar{\Delta \mathrm{FA}}$ are specified. For both acquisition schemes, deviations from the linear assumption increase with higher reference voltages. However, the assumption holds better for the interleaved scheme, with consistently lower mean and maximum FA errors.
**Figure S5.** Mean signals over the reference voltage used for the simulation of the sequential and interleaved acquisition for the phantom (A), heart (B) and prostate (C). Mean signals were calculated over all transmit channels and over all voxels contained within the corresponding ROIs. In general, the mean signals increase with higher reference voltage up to a certain point, as visible in the simulations of the prostate. While the course of the mean signals is similar for both acquisition schemes, slightly higher values are obtained with the interleaved acquisition scheme.
**Figure S6.** Ground truth $B_{1}^{+}$ distributions and relative $B_{1}^{+}$ magnitudes for the heart (a, Tx1) and prostate (b, Tx5) obtained from simulations of the sequential and interleaved acquisition schemes for different reference voltages. The ground truth $B_{1}^{+}$ data is based on electromagnetic field simulations of the Duke body model. At low reference voltages, the simulations show a good match to the ground truth for both organs and both acquisition schemes. However, for increasing voltages, strong deviations for the relative $B_{1}^{+}$ maps of the sequential acquisition scheme become visible. For the interleaved acquisition scheme, deviations appear less severe and in the case of the prostate are hardly visible even for the highest simulated reference voltage.
**Figure S7.** (A) Single channel transmit efficiencies obtained with an AFI sequence for a 4Tx/16Rx 7 T breast coil (Rapid Biomedical GmbH, Rimpar, Germany). The phantom was filled with a PVP solution (49% polyvinylpyrrolidone (PVP), 49.2% distilled water, 1.8% NaCl) with a T_1_ time of 534 ms. (B) Normalized mean FA errors ($\bar{∆\mathrm{FA}}$) over reference voltages obtained for simulations of the sequential and interleaved acquisition scheme based on the transmit efficiencies of the 4Tx/16Rx breast coil. The evaluation of the signal linearity shows similar results to the 8Tx/16Rx body array, with lower $\bar{∆\mathrm{FA}}$ values for the interleaved acquisition scheme in all simulated cases. (C) Mean signals over $\bar{∆\mathrm{FA}}$ for the sequential and interleaved acquisition schemes obtained for the simulations performed with the 4Tx/16Rx breast coil. For the same level of $\bar{∆\mathrm{FA}}$, the interleaved acquisition scheme results in a higher mean signal than the sequential acquisition scheme.
**Figure S8.** Normalized mean FA errors ($\bar{∆\mathrm{FA}}$) for the sequential and interleaved acquisition schemes for steady‐state simulations based on the phantom data with a reference voltage of 75 V (A) and the heart data with a reference voltage of 150 V (B). The simulations differed in the order that the individual transmit channels were operated, with the specific permutation given in the first column. Since the steady‐state simulations of the sequential acquisition scheme are done independently for each transmit channels, the $\bar{∆\mathrm{FA}}$ values are identical for all permutations. For the simulation of the interleaved acquisition scheme, different permutations of the Tx channels result in very small differences between the steady‐state $\bar{∆\mathrm{FA}}$ values.
**Figure S9.** Evaluation of the normalized mean FA error for different combinations of U_ref_ and T_1_ for the sequential acquisition scheme (A), the interleaved acquisition scheme (B) and the difference between the errors of both acquisition schemes (C). The evaluation was performed for simulations with the $B_{1}^{+}$ distribution of the disk phantom for a fixed TR of 4.5 ms but assuming different T_1_ times of the contained solution. As expected, lower T_1_ times lead to lower errors for both acquisition schemes, as the linear FA range increases due to a diminishing T_1_ bias. Notably, errors are consistently lower for the interleaved than for the sequential acquisition scheme. Even for a relatively low T_1_ time of 500 ms, a high reference voltage (150 V) leads to considerable differences in the errors between both acquisition schemes (${\bar{\Delta \mathrm{FA}}}_{\mathrm{Seq}}$ = 17.2%, ${\bar{\Delta \mathrm{FA}}}_{\mathrm{IL}}$ = 5.8%).
