# Supplementary material for: Evaluation of an interleaved acquisition scheme for improved robustness of channel‐wise relative B1 + mapping at 7 T
Source: Magn Reson Med. 2025 Sep 29;95(2):1157–68. doi: 10.1002/mrm.70101 (PMC12681310; doi:10.1002/mrm.70101)
Supplement: Supplementary file 1 — Figure S1. (A) Structure of the 8Tx/16Rx body array used in this work. The RF array consists of an upper and a lower part with 4Tx/8Rx channels each. In the transmit case, two of the receive elements (numbered 1–16) are combined to one transmit element (colored). (B) Illustration and measurement setup of the phantom used in this work. The phantom consists of two disks filled with 2 L of a NaCl solution and two disks filled with 2 L of oil (height ∼35 mm each, diameter ∼240 mm). For the measurement, the disks are stacked on top of each other, with the two NaCl disks on the outside and the oil disks positioned between them. Note that for the simulations and evaluations performed in this work, only the data from the NaCl disks were analyzed. Figure S2. Channel‐wise relative absolute errors between the phantom simulations of the sequential (left) and interleaved (right) acquisition schemes and the AFI ground‐truth (GT) for a reference voltage of 150 V. The errors are given relative to the FA of the GT and are depicted for a transversal and coronal slice (indicated by white dashed line). For the sequential acquisition scheme, errors are highest in areas close to the transmitting element, whereas for the interleaved scheme the errors distribution appears similar between different transmit channels. In general, errors are considerably higher for the sequential acquisition scheme. Figure S3. (A) AFI ground‐truth (GT) and corresponding relative B1+ maps for Tx1, acquired with the sequential and interleaved acquisition schemes for three different reference voltages in an in vivo measurement of the heart. The B1+ maps were normalized to the maximum within the slice to ensure consistent windowing and a 3D mask was generated based on the AFI measurement. (B) Correlation factors between the AFI GT and the acquired relative B1+ maps based on all data points within the 3D mask volume. For the lowest reference voltage of 50 V the relative B1+ maps of both acquisition scheme show a g [file MRM-95-1157-s001.docx]

## Supporting Information

**Evaluation of an interleaved acquisition scheme for improved robustness of channel-wise relative** $\mathbf{B}_{\mathbf{1}}^{\mathbf{+}}$ **mapping at 7T**

Nico Egger^1^, Laurent Ruck^1^, Sophia Nagelstraßer^1^, Judith Schirmer^1^, Saskia Wildenberg^1,2^, Andreas K. Bitz^2^, Jürgen Herrler^3^, Sebastian Schmitter^4,5,6^, Michael Uder^1^, Armin M. Nagel^1,6^

^1^ *Institute of Radiology, University Hospital Erlangen, Friedrich-Alexander-Universität Erlangen-Nürnberg (FAU), Erlangen, Germany*

*^2^ Electrical Engineering and Information Technology, University of Applied Sciences - FH Aachen, Aachen, Germany*

*^3^ Siemens Healthcare GmbH, Erlangen, Germany*

*^4^ Physikalisch-Technische Bundesanstalt (PTB), Braunschweig and Berlin, Germany*

*^5^* *Center for Magnetic Resonance Research, University of Minnesota, Minneapolis, Minnesota USA*

^6^ *Division of Medical Physics in Radiology, German Cancer Research Centre (DKFZ), Heidelberg, Germany*


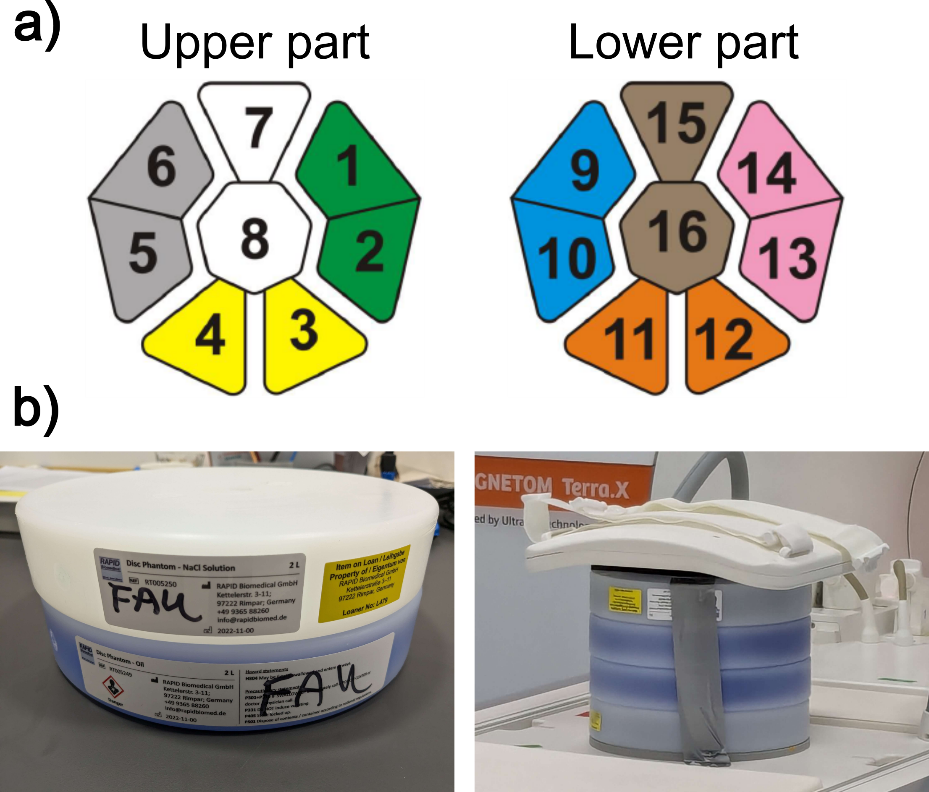


Figure S1: (a) Structure of the 8Tx/16Rx body array used in this work. The RF array consists of an upper and a lower part with 4Tx/8Rx channels each. In the transmit case, two of the receive elements (numbered 1-16) are combined to one transmit element (colored). (b) Illustration and measurement setup of the phantom used in this work. The phantom consists of two disks filled with 2L of a NaCl solution and two disks filled with 2L of oil (height ∼35mm each, diameter ∼240mm). For the measurement, the disks are stacked on top of each other, with the two NaCl disks on the outside and the oil disks positioned between them. Note that for the simulations and evaluations performed in this work, only the data from the NaCl disks were analyzed.


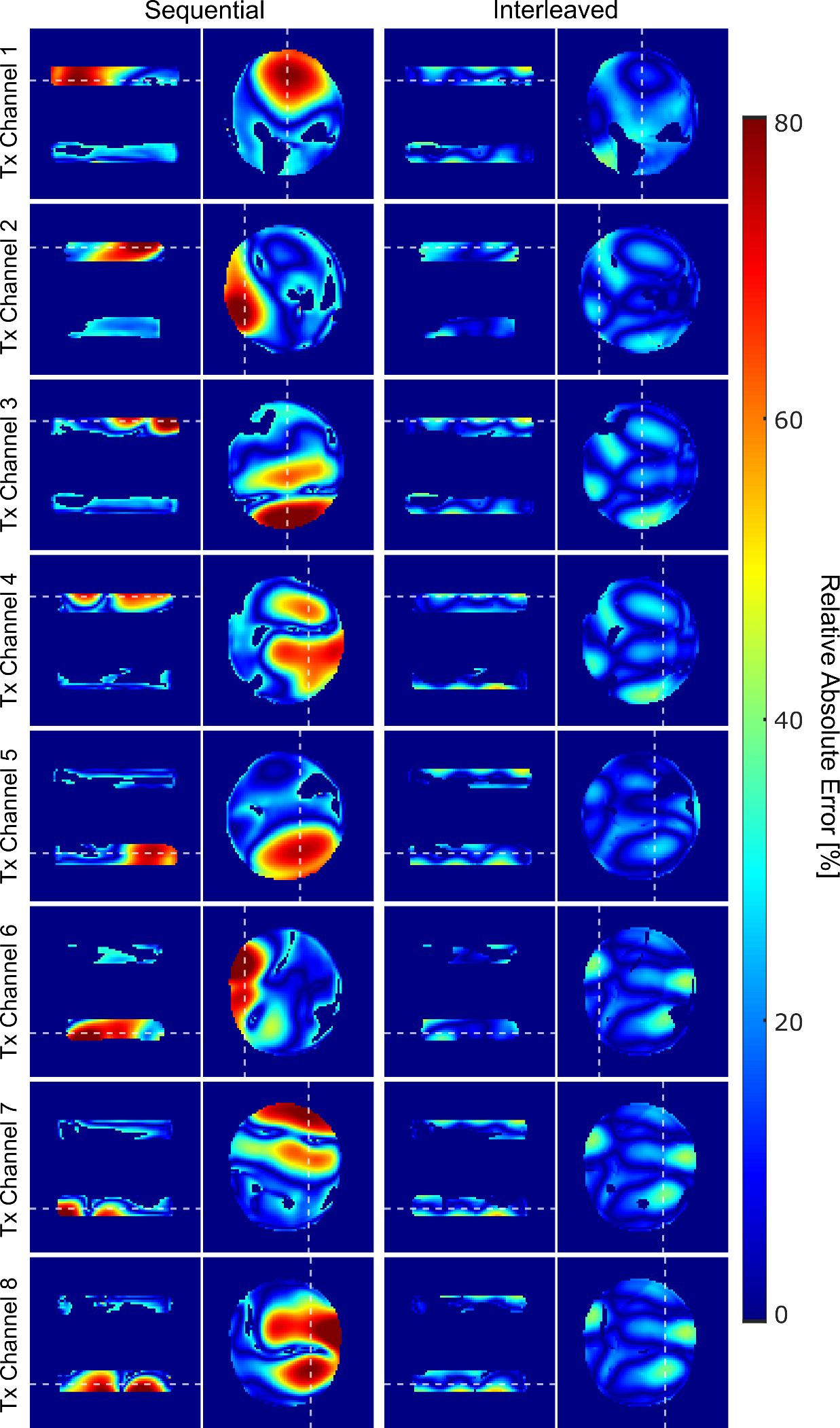


Figure S2: Channel-wise relative absolute errors between the phantom simulations of the sequential (left) and interleaved (right) acquisition schemes and the AFI ground-truth (GT) for a reference voltage of 150V. The errors are given relative to the FA of the GT and are depicted for a transversal and coronal slice (indicated by white dashed line). For the sequential acquisition scheme, errors are highest in areas close to the transmitting element, whereas for the interleaved scheme the errors distribution appears similar between different transmit channels. In general, errors are considerably higher for the sequential acquisition scheme.


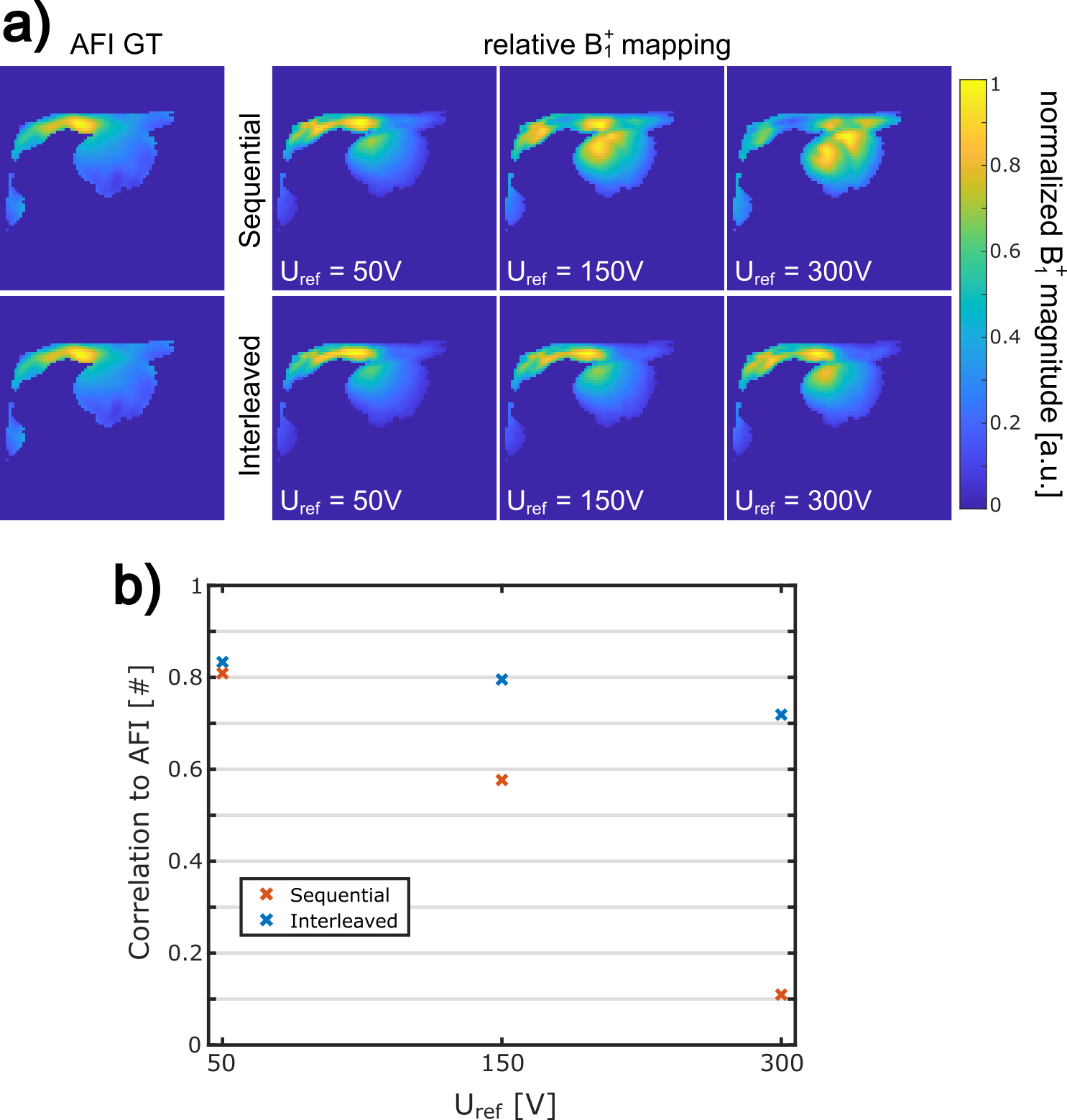


Figure S3: (a): AFI ground-truth (GT) and corresponding relative $B_{1}^{+}$ maps for Tx1, acquired with the sequential and interleaved acquisition schemes for three different reference voltages in an in vivo measurement of the heart. The $B_{1}^{+}$ maps were normalized to the maximum within the slice to ensure consistent windowing and a 3D mask was generated based on the AFI measurement. (b): Correlation factors between the AFI GT and the acquired relative $B_{1}^{+}$ maps based on all data points within the 3D mask volume. For the lowest reference voltage of 50V the relative $B_{1}^{+}$ maps of both acquisition scheme show a good qualitative (a) and quantitative (b) agreement with the GT. With increasing voltage, the correlation only decreases moderately for the interleaved scheme, whereas it drops sharply for the sequential scheme. Correspondingly, the qualitative evaluation shows large deviations to the GT at 300V for the sequential scheme, whereas a better match is observed for the interleaved scheme. Relative $B_{1}^{+}$ sequence parameters as described in section 2.5, with 10000 projections. AFI parameters were: TR1/TR2/TE = 15/75/3.03ms, nominal FA = 65°, resolution = (4mm)^3^, projections = 5000, acquisition time = 7:30min.


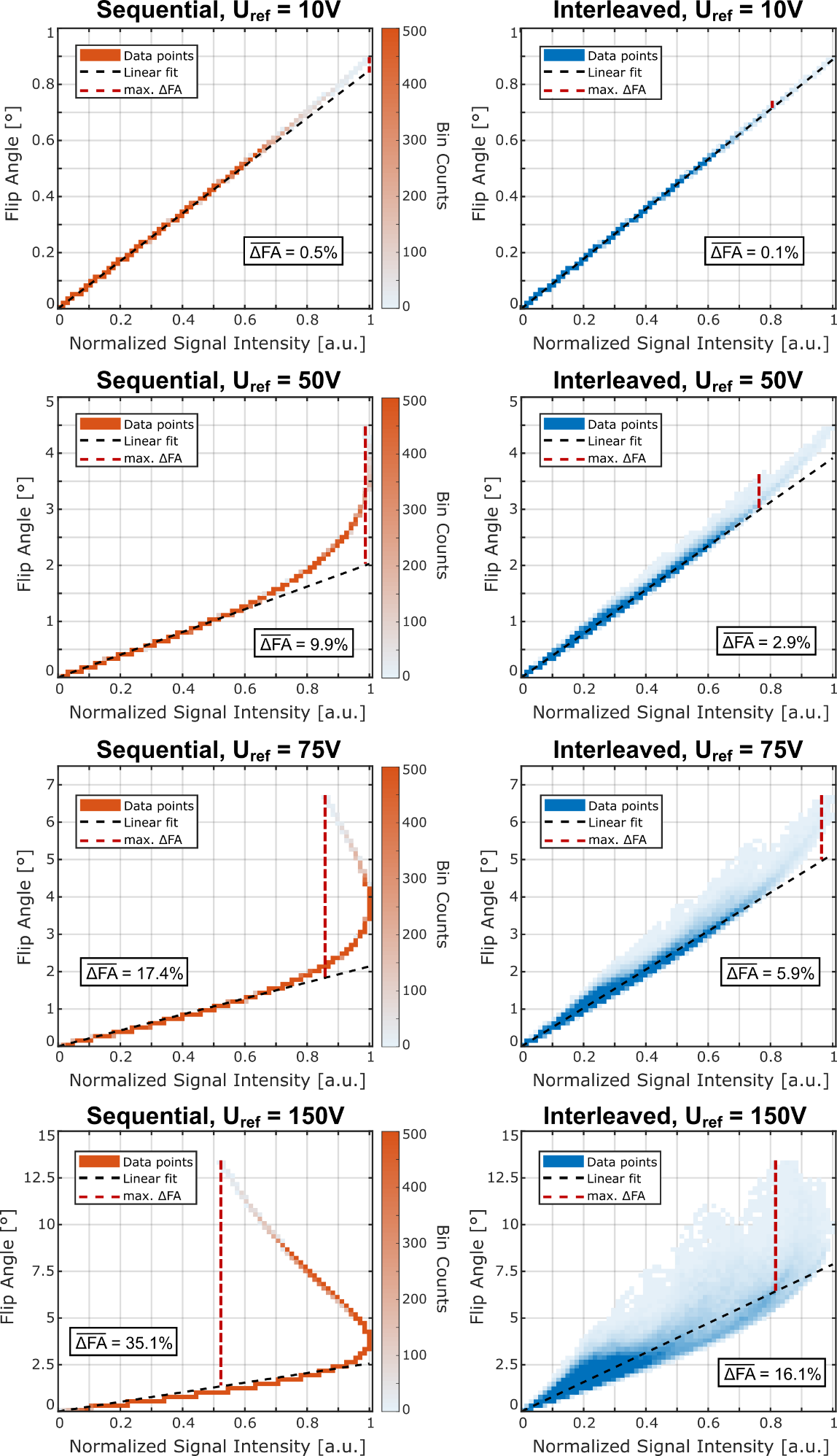


Figure S4: Extension of the scatter plots shown in Figure 3 for four additional reference voltages. The plots depict histograms of the data point distribution of the ground-truth FA over the normalized signal intensity resulting from simulations of the phantom with the sequential (left) and interleaved (right) acquisition schemes. In addition, the maximum FA deviations are depicted and the normalized mean FA errors $\bar{\Delta FA}$ are specified. For both acquisition schemes, deviations from the linear assumption increase with higher reference voltages. However, the assumption holds better for the interleaved scheme, with consistently lower mean and maximum FA errors.


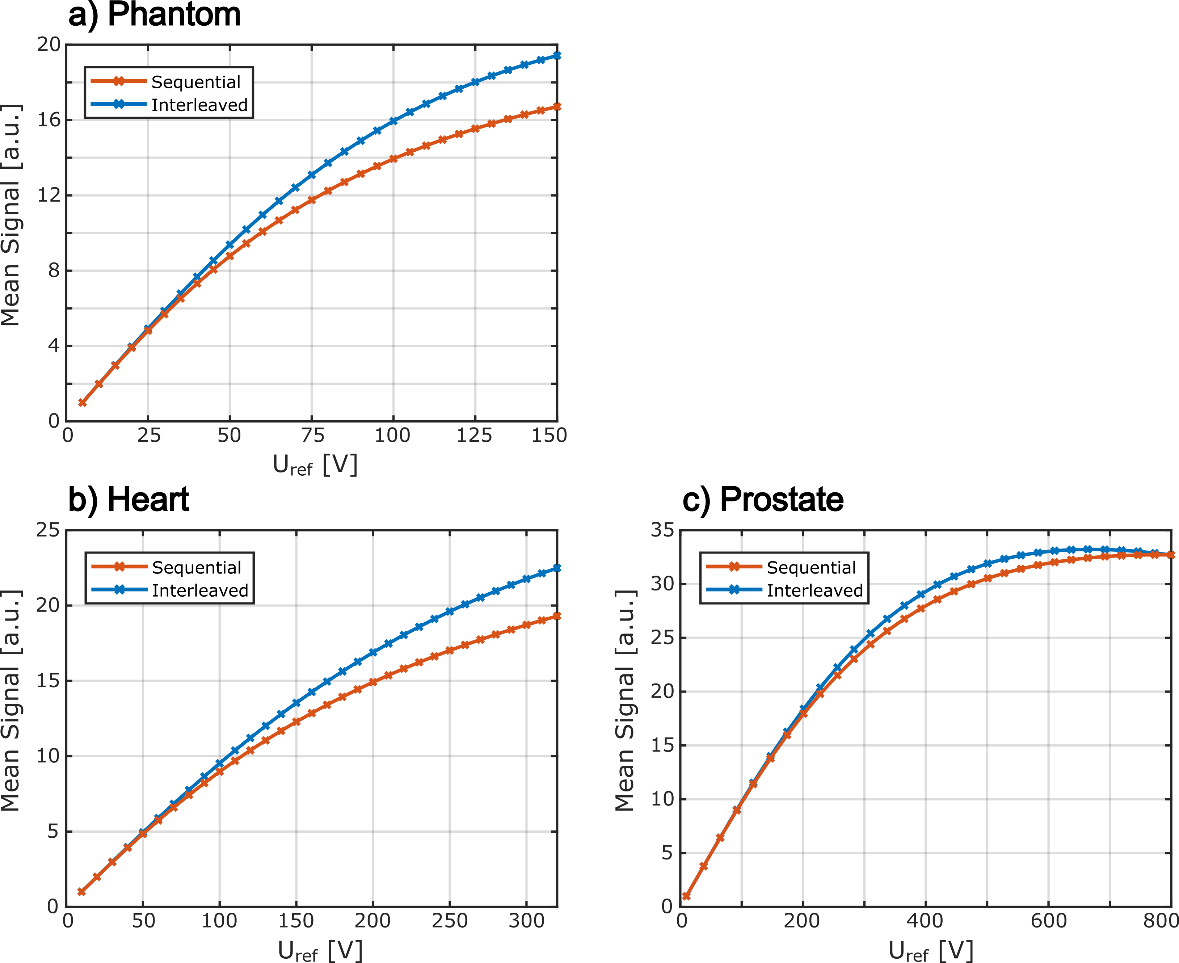


Figure S5: Mean signals over the reference voltage used for the simulation of the sequential and interleaved acquisition for the phantom (a), heart (b) and prostate (c). Mean signals were calculated over all transmit channels and over all voxels contained within the corresponding ROIs. In general, the mean signals increase with higher reference voltage up to a certain point, as visible in the simulations of the prostate. While the course of the mean signals is similar for both acquisition schemes, slightly higher values are obtained with the interleaved acquisition scheme.


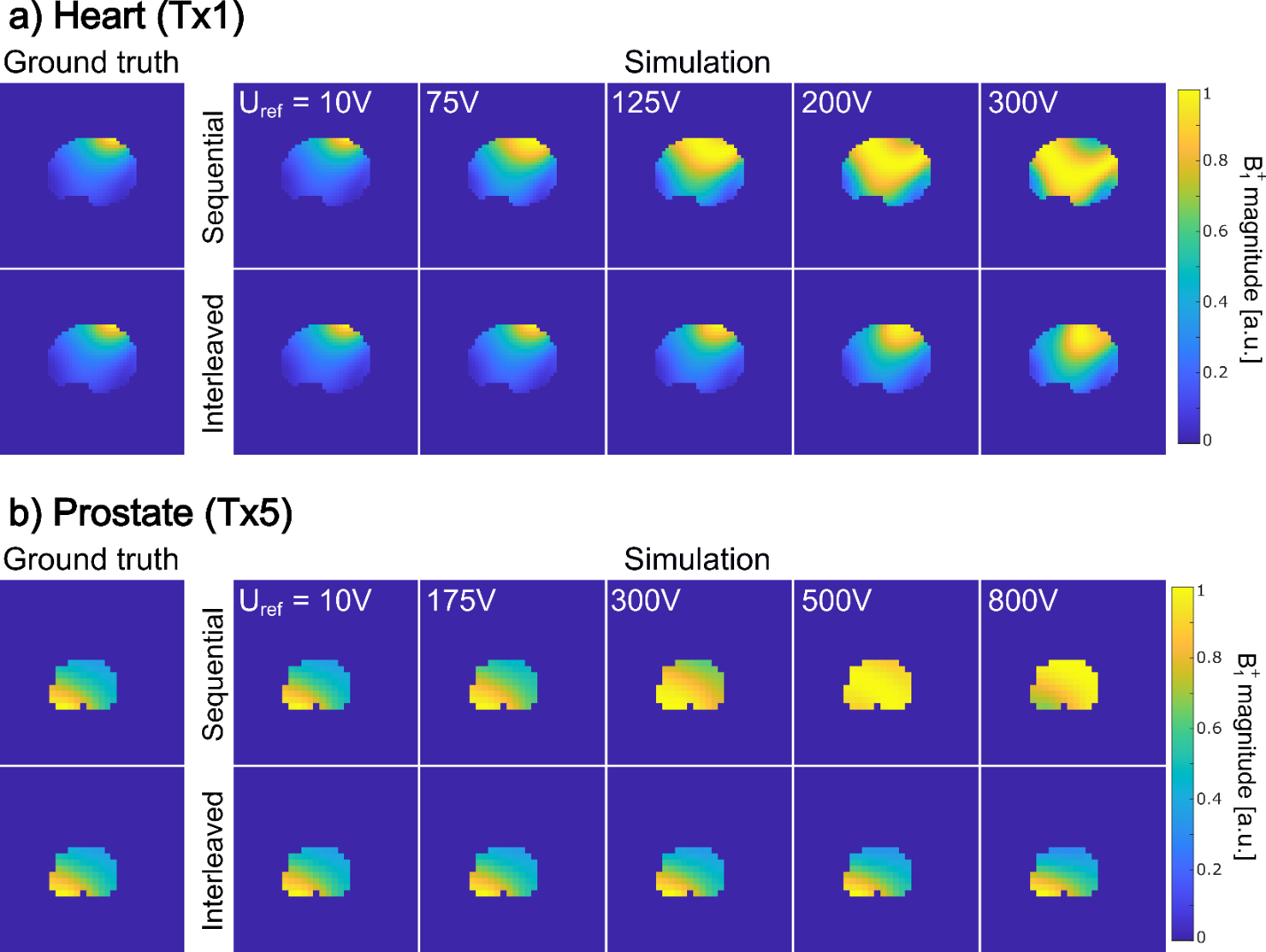


Figure S6: Ground truth $B_{1}^{+}$ distributions and relative $B_{1}^{+}$ magnitudes for the heart (a, Tx1) and prostate (b, Tx5) obtained from simulations of the sequential and interleaved acquisition schemes for different reference voltages. The ground truth $B_{1}^{+}$ data is based on electromagnetic field simulations of the Duke body model. At low reference voltages, the simulations show a good match to the ground truth for both organs and both acquisition schemes. However, for increasing voltages, strong deviations for the relative $B_{1}^{+}$ maps of the sequential acquisition scheme become visible. For the interleaved acquisition scheme, deviations appear less severe and in the case of the prostate are hardly visible even for the highest simulated reference voltage.


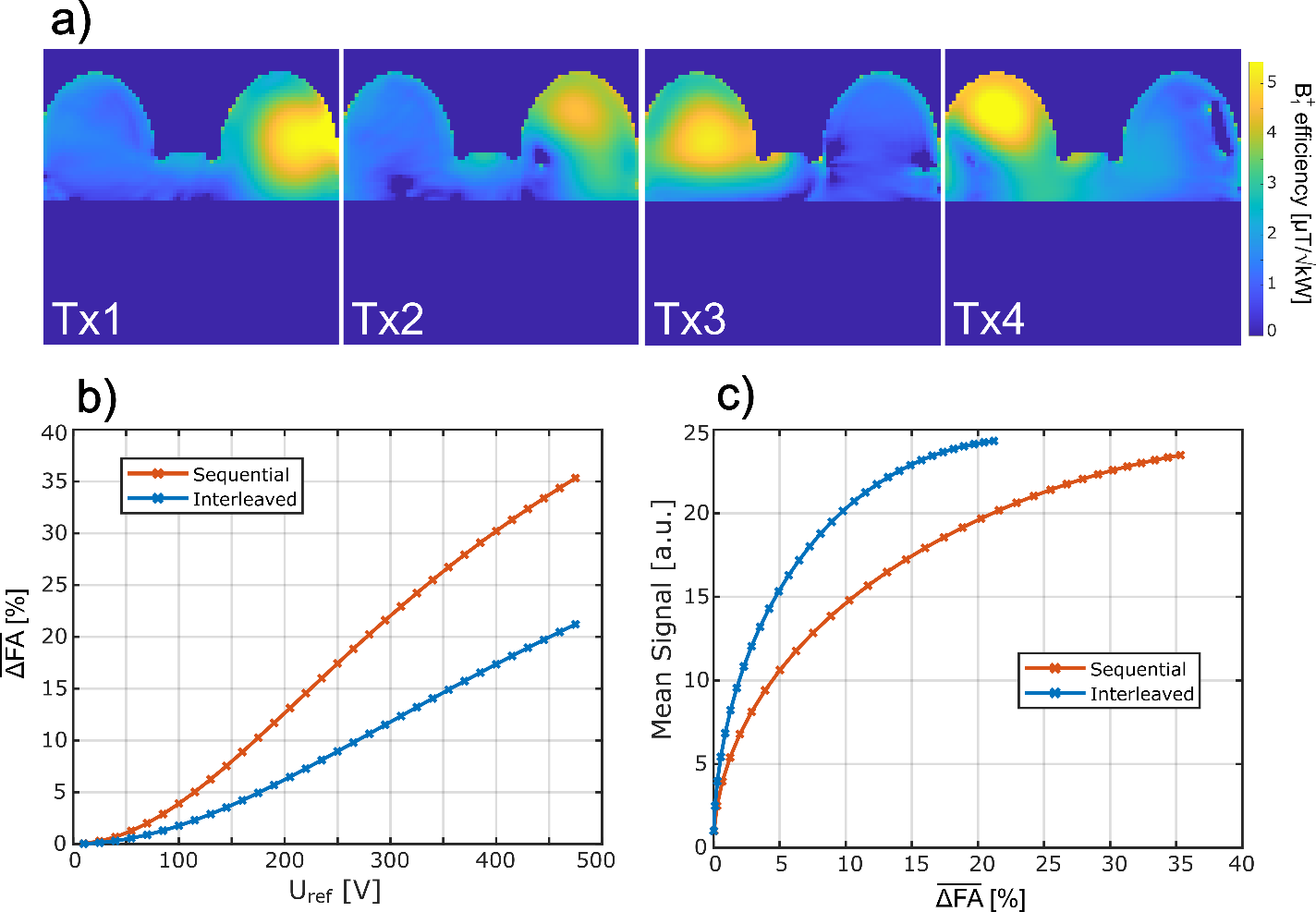


Figure S7: (a) Single channel transmit efficiencies obtained with an AFI sequence for a 4Tx/16Rx 7T breast coil (Rapid Biomedical GmbH, Rimpar, Germany). The phantom was filled with a PVP solution (49% polyvinylpyrrolidone (PVP), 49.2% distilled water, 1.8% NaCl) with a T_1_ time of 534ms. (b) Normalized mean FA errors ($\bar{\Delta FA}$) over reference voltages obtained for simulations of the sequential and interleaved acquisition scheme based on the transmit efficiencies of the 4Tx/16Rx breast coil. The evaluation of the signal linearity shows similar results to the 8Tx/16Rx body array, with lower $\bar{\Delta FA}$ values for the interleaved acquisition scheme in all simulated cases. (c) Mean signals over $\bar{\Delta FA}$ for the sequential and interleaved acquisition schemes obtained for the simulations performed with the 4Tx/16Rx breast coil. For the same level of $\bar{\Delta FA}$, the interleaved acquisition scheme results in a higher mean signal than the sequential acquisition scheme.


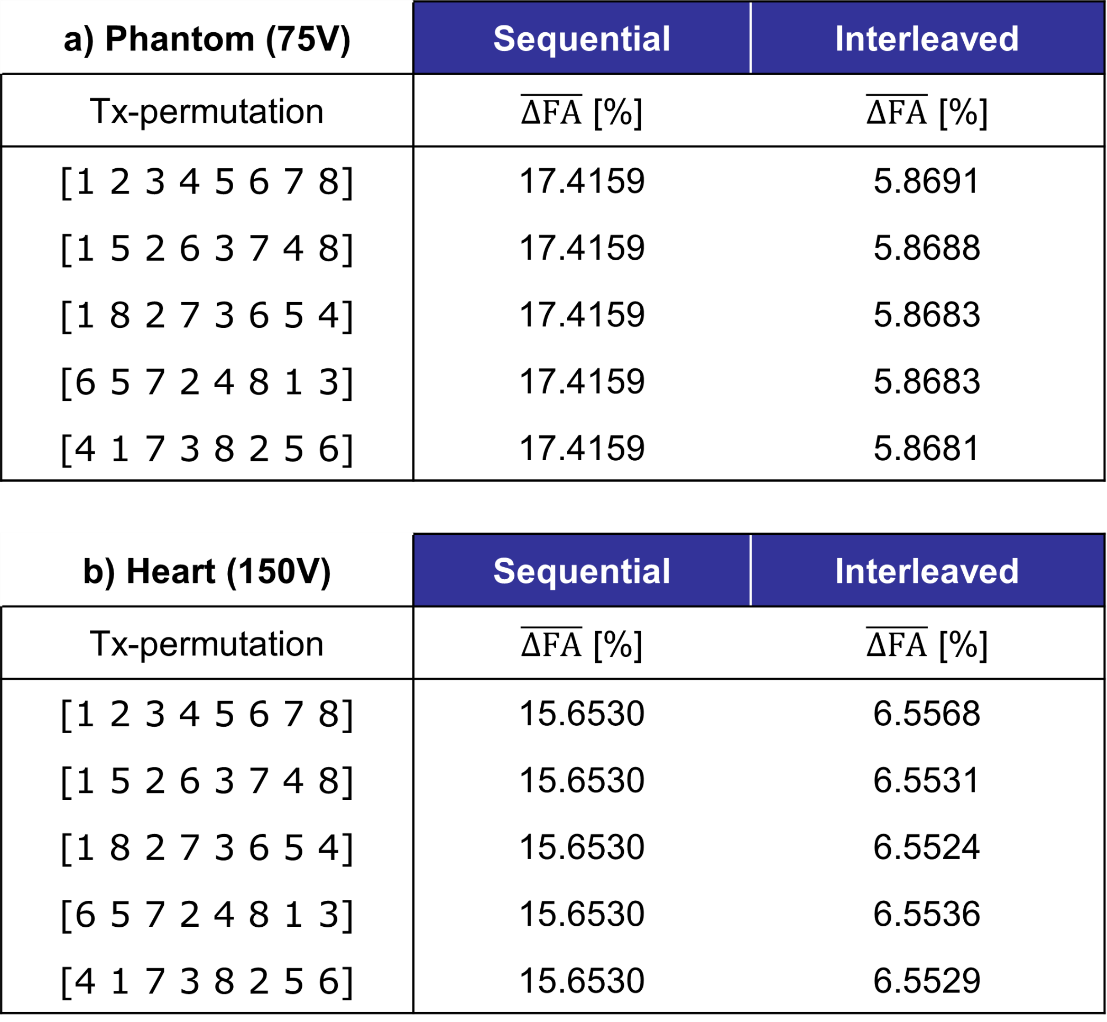


Figure S8: Normalized mean FA errors ($\bar{\Delta FA}$) for the sequential and interleaved acquisition schemes for steady-state simulations based on the phantom data with a reference voltage of 75V (a) and the heart data with a reference voltage of 150V (b). The simulations differed in the order that the individual transmit channels were operated, with the specific permutation given in the first column. Since the steady-state simulations of the sequential acquisition scheme are done independently for each transmit channels, the $\bar{\Delta FA}$ values are identical for all permutations. For the simulation of the interleaved acquisition scheme, different permutations of the Tx channels result in very small differences between the steady-state $\bar{\Delta FA}$ values.


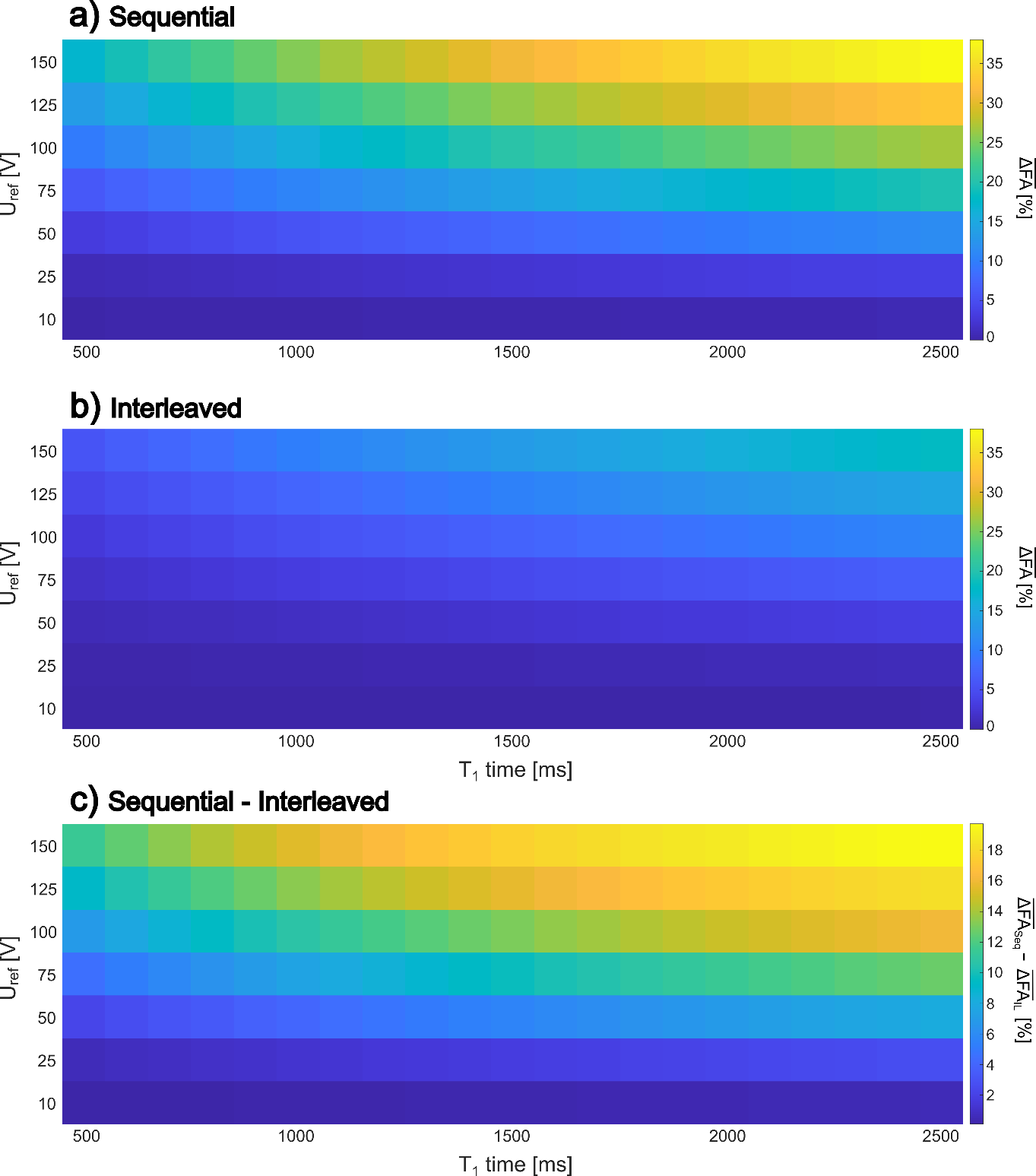


Figure S9: Evaluation of the normalized mean FA error for different combinations of U_ref_ and T_1_ for the sequential acquisition scheme (a), the interleaved acquisition scheme (b) and the difference between the errors of both acquisition schemes (c). The evaluation was performed for simulations with the $B_{1}^{+}$ distribution of the disk phantom for a fixed TR of 4.5ms but assuming different T_1_ times of the contained solution. As expected, lower T_1_ times lead to lower errors for both acquisition schemes, as the linear FA range increases due to a diminishing T_1_ bias. Notably, errors are consistently lower for the interleaved than for the sequential acquisition scheme. Even for a relatively low T_1_ time of 500ms, a high reference voltage (150V) leads to considerable differences in the errors between both acquisition schemes ($\bar{\Delta FA}_{Seq}$ = 17.2%, $\bar{\Delta FA}_{IL}$ = 5.8%).
